# Excess mortality among patients with severe mental disorders and effects of community-based mental healthcare: A community-based prospective study in Sichuan, China – RETRACTION

**DOI:** 10.1192/bjo.2024.38

**Published:** 2024-03-18

**Authors:** Yaxi Li, Lijing L. Yan, Carine Ronsmans, Hong Wen, Jiajun Xu, Dan Wang, Min Yang

**Keywords:** Severe mental disorder, excess mortality, community-based mental health care, high-risk behaviour, medication adherence, retraction

We, the Editors of BJPsych Open, have retracted the following article:

Li, Y., Yan, L., Ronsmans, C., Wen, H., Xu, J., Wang, D., & Yang, M. (2021). Excess mortality among patients with severe mental disorders and effects of community-based mental healthcare: A community-based prospective study in Sichuan, China. *BJPsych Open*, 7(3), E84. doi:10.1192/bjo.2021.46

In November 2022, the authors of this paper voluntarily submitted a corrigendum to the publisher which acknowledged an error in Supplementary Table 2.

An investigation by senior members of the Editorial Board revealed that whilst this was a relatively minor “cut and paste” error, further changes would be needed throughout the main manuscript to rectify the error. The investigation also indicated further areas for the authors to address.

To ensure research integrity, these senior members decided that retraction of the original article with subsequent resubmission of a corrected paper would be the best course of action.

No institutional investigation or sanctioning by the Journal is indicated.

YL, LY and MY have confirmed agreement to the retraction. CR, HW, JX and DW could not be reached.
